# Barriers, facilitators, perceptions and impact of interventions in implementing antimicrobial stewardship programs in hospitals of low-middle and middle countries: a scoping review

**DOI:** 10.1186/s13756-024-01369-6

**Published:** 2024-01-23

**Authors:** Md. Golam Dostogir Harun, Shariful Amin Sumon, Istiaque Hasan, Fairoze Masuda Akther, Md. Saiful Islam, Md. Mahabub Ul Anwar

**Affiliations:** 1 Infectious Diseases Division, icddr, b, 68 Shaheed Tajuddin Ahmed Sarani, Mohakhali, Dhaka, 1212 Bangladesh; 2 University of New South Wales, Sydney, Australia; 3 Department of Population Sciences, University of Dhaka, Dhaka, Bangladesh

**Keywords:** Antimicrobial stewardship program (ASP), Scoping review, Low- and middle-income countries (LMIC)

## Abstract

**Background:**

Antimicrobial stewardship programs (ASPs) are pivotal components of the World Health Organization's Global Action Plan to combat antimicrobial resistance (AMR). ASPs advocate rational antibiotic usage to enhance patient-centered outcomes. However, existing evidence on ASPs and their determinants is largely limited to well-equipped hospitals in high-income nations.

**Objective:**

This scoping review aimed to examine the current state of hospital-based ASPs in low- and middle-income countries (LMICs), shedding light on barriers, facilitators, prescribers’ perceptions and practices, and the impact of ASP interventions.

**Design:**

Scoping review on ASP.

**Methods:**

Adhering to PRISMA guidelines, we conducted electronic database searches on PubMed, Scopus, and Google Scholar, covering ASP articles published between January 2015 and October 2023. Our review focused on four key domains: barriers to ASP implementation, facilitators for establishing ASP, ASP perceptions and practices of prescribers, and the impact of ASP interventions. Three reviewers separately retrieved relevant data from the included citations using EndNote 21.0.

**Results:**

Among the 7016 articles searched, 84 met the inclusion criteria, representing 34 LMICs. Notably, 58% (49/84) of these studies were published after 2020. Barriers to ASP implementation, including human-resources shortage, lack of microbiology laboratory support, absence of leadership, and limited governmental support, were reported by 26% (22/84) of the studies. Facilitators for hospital ASP implementation identified in five publications included the availability of antibiotic guidelines, ASP protocol, dedicated multidisciplinary ASP committee, and prompt laboratory support. The majority of the research (63%, 53/84) explored the impacts of ASP intervention on clinical, microbiological, and economic aspects. Key outcomes included increased antibiotic prescription appropriateness, reduced antimicrobial consumption, shorter hospital stays, decreased mortality rate, and reduced antibiotic therapy cost.

**Conclusions:**

The published data underscores the imperative need for widespread antimicrobial stewardship in LMIC hospital settings. Substantial ASP success can be achieved through increasing human resources, context-specific interventions, the development of accessible antibiotic usage guidelines, and heightened awareness via training and education.

**Supplementary Information:**

The online version contains supplementary material available at 10.1186/s13756-024-01369-6.

## Background

Antimicrobial resistance (AMR) has emerged as one of the top ten global public health threats of the twenty-first century according to the World Health Organization (WHO) [1, 2]. In 2019, approximately 1.3 million deaths were directly attributable to AMR [3, 4]. If no measures are taken, it is projected that by 2050 AMR could lead to the deaths of 10 million people annually, with up to 90% of these fatalities occurring in low- and middle-income countries (LMICs) [4]. In addition to the health consequences, AMR also carries significant financial implications at both patient and societal levels. According to a World Bank projection, AMR could reduce gross domestic product (GDP) by 1.1–3.8% by 2050, necessitating an annual investment of US$9 billion to counteract AMR effectively [5, 6].

The primary drivers of AMR in LMICs include the absence of antibiotic guidelines to regulate prescribing practices [7], irrational use of antibiotics [8], the financial incentive of prescribers, easy accessibility and ‘over the counter access’ of antibiotics, self-medication, patient pressure, lack of sanitation, poor infection prevention and control (IPC) practices, and the lack of an antimicrobial stewardship program (ASP) [9]. To address AMR, WHO formulated a global action plan (GAP) in 2015 [10] which identified ASP as a cornerstone to curtail the inappropriate use of antibiotics for therapeutic use [11, 12]. Antibiotic stewardship involves a set of coordinated actions that promote the appropriate use of antimicrobials through evidence-based, multidisciplinary interventions against AMR [12, 13]. In hospital settings, along with infection control measures, ASPs are considered a fundamental strategy to limit the emergence and escalation of AMR [14], improve clinical outcomes, and reduce healthcare costs by promoting the rational use of antibiotics [15, 16].

ASP features may vary [17] but typically include a range of interventions tailored to fit the hospital’s infrastructure [18]. Stewardship interventions can be categorized as persuasive (education and feedback), structural (introduction of new diagnostic tests to guide antibiotic treatment), enabling (guidelines on antibiotic use), or restrictive (expert approval for the use of certain antibiotics) [2, 16, 19]. The key components identified in successful ASPs include leadership commitment, drug expertise, prescribers’ accountability, and orientation training for prescribers [13]. Most of the recent evidence on ASP comes from resource-intensive hospitals in high-income countries [20, 21], making it uncertain whether these findings apply to resource-constrained hospitals [22, 23].

This scoping review aimed to map and summarize published data on ASPs deployed in hospital settings in LMICs. It focuses on sequentially integrating four key domains: barriers to implementing ASP, facilitators to establishing ASP, ASP perceptions and practices of prescribers, and the impact of ASP interventions in LMICs. Prior reviews on LMICs mostly focused on the impact of ASP interventions or the ASP methods most widely employed in hospital settings [10, 15, 24]. The findings from this review would be valuable for key stakeholders and policymakers to get an insight into the common obstacles encountered in hospital settings across LMICs, and the key areas on which they need to focus prior to ASP implementation. The review will also help the stakeholders get an idea of where we stand in terms of prescribers’ knowledge and perception of ASP across LMIC.

## Methods

### Search strategy

This scoping review adheres to the Preferred Reporting Items for Systematic Reviews and Meta-Analysis extension for scoping reviews (PRISMA-ScR) guidelines [25]. We developed a systematic search strategy using keywords and medical subject headings (MeSH) terms to identify relevant articles published between January 2015 and December 2023 in MEDLINE, Scopus, and Google Scholar databases. Our search focused on articles related to ASP published after the WHO GAP Plan on AMR in 2015 [13], as this led to increased hospital-based ASP publications in LMICs [10, 24]. We only included articles published in the English language. The review encompassed countries classified as low, and lower-middle income, according to the World Bank classification system [26].

### Study inclusion criteria

Based on our research questions, we established the following inclusion and exclusion criteria to select relevant articles.

Inclusion criteria

- ASP interventions for adult patients conducted in hospital settings in LMICs
- Articles, abstracts, poster presentations, preprints, and grey literature
- Antimicrobial or antibiotic stewardship in the human population
- Published articles limited to the English language

Exclusion criteria

- Case studies, narrative reviews, editorials, discussion articles, conference papers, invited articles, special reports on ASP
- Reviews, commentaries, or expert opinions on ASP
- ASP in animal, agriculture, or community settings
- Articles focusing solely on the use of antifungal or antiviral stewardships

Our search strategy employed a combination of first, second, and third-string terms. The first string included keywords such as ‘antimicrobial’ ‘antibiotic’ ‘antimicrobial resistance’ and ‘antibiotic resistance.’ The second string is composed of keywords related to antimicrobial stewardship, such as ‘stewardship’ ‘formulary restriction’ and ‘prospective audit.’ The third string included the terms ‘low-middle income countries’ ‘less developed countries’ ‘underdeveloped’ and ‘developing nation.’ These search terms were combined using Boolean operators “OR” or “AND” to refine search results. Detailed search strategies can be found in the supplementary file (Additional file 1: Appendix 1. Search strategy).

### Study selection

Three reviewers (MGDH, SAS, IH) conducted a systematic title screening of the databases using the specified keywords. Duplicate records were removed and then imported into a citation management program (EndNote 21.0). IH and SAS independently assessed titles and abstracts, while MGDH reviewed the final list. Subsequently, full texts of potentially relevant articles were obtained and evaluated for eligibility.

### Data extraction

We extracted and organized data into four domains listed below:

- Domain 1: Barriers to ASP implementation
- Domain 2: Facilitators for establishing ASP
- Domain 3: ASP perceptions and practices of prescribers (physicians or pharmacists)
- Domain 4: Impact of ASP interventions

The retrieved data included the name of the first author, year of publication, place of study, study design, setting, type of hospital (public, private, or university), study population, and measured outcomes (Table 1). Barriers to ASP implementation (Domain 1, Table 2) and facilitators for establishing ASP (Domain 2, Table 3) were categorized based on constraints and contributing factors described in the articles. The identified impediments or lack of facilities or resources for ASP implementation from the mentioned studies were assembled in Domain 1 as barriers, whereas the availability of facilities or resources to execute ASP was considered in Domain 2 as facilitators. Physicians’ perception and practices related to ASP were categorized into knowledge, attitude, and practices (KAP) (Domain 3, Table 4). The impact of ASP interventions was described into five subcategories (Domain 4, Table 5): (1) antibiotic prescription by prescribers (physicians or pharmacists), (2) antibiotic consumption (defined daily doses or days on therapy), (3) clinical outcomes (e.g., length of hospital stay, in-hospital mortality), (4) microbiological outcomes (multi-drug resistance, bacterial resistance patterns), and (5) economic outcomes (hospital antibiotic procurement cost, cost of antibiotic therapy). Antibiotic prescription refers to the quality of antibiotic use (proportion of prescriptions with inappropriate antibiotic use, inappropriate use without clinical indications, unnecessary double coverage, incorrect frequency, dosage, timing, broad-spectrum and expensive antibiotics when inexpensive and narrow-spectrum alternatives were available), which was prescribed by hospital physicians or pharmacists.

**Table 1:**
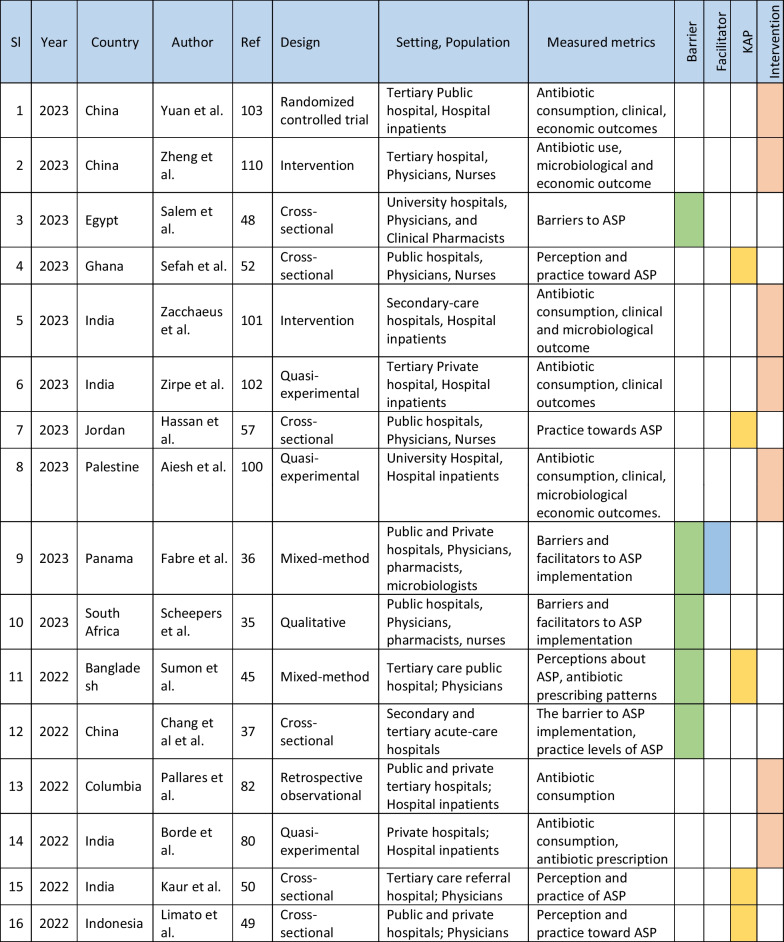

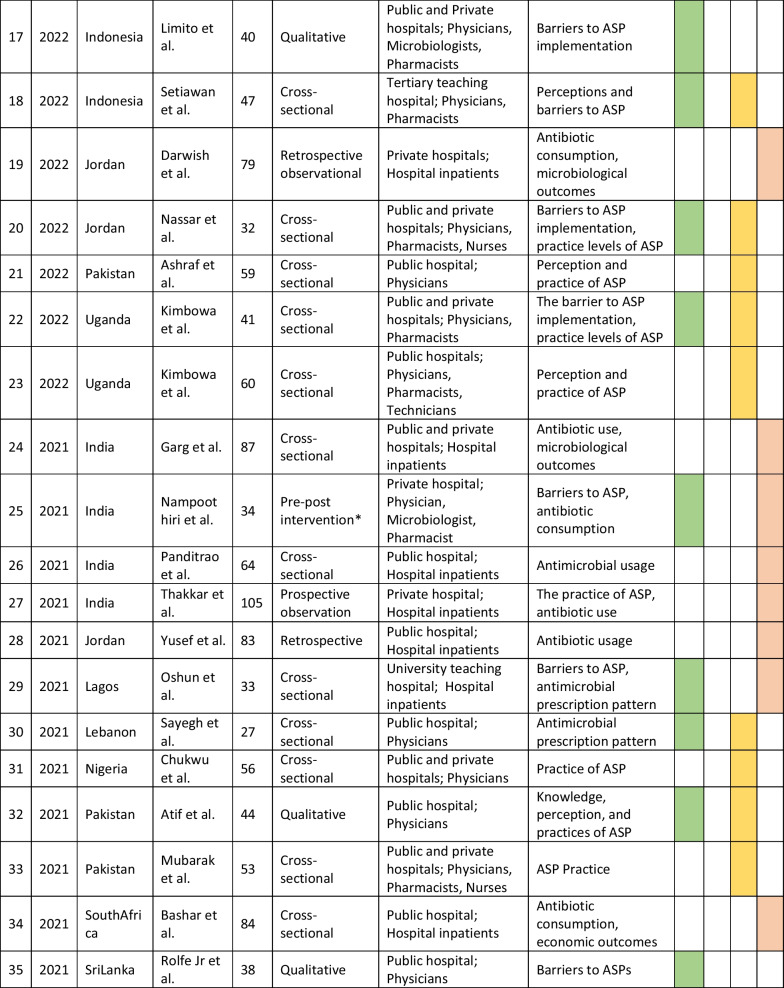

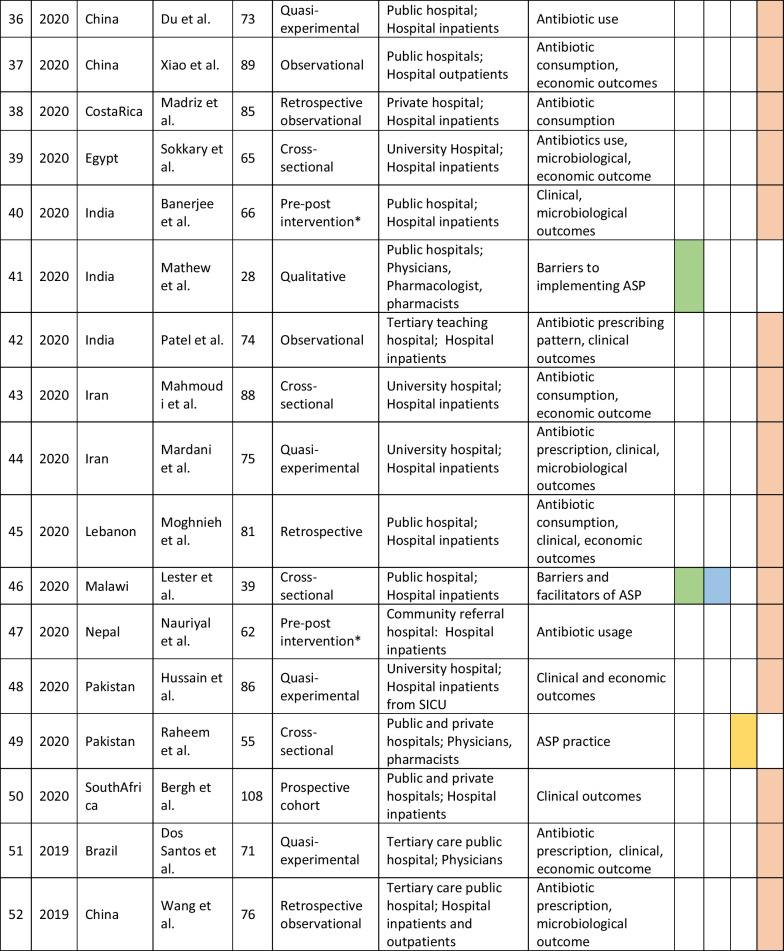

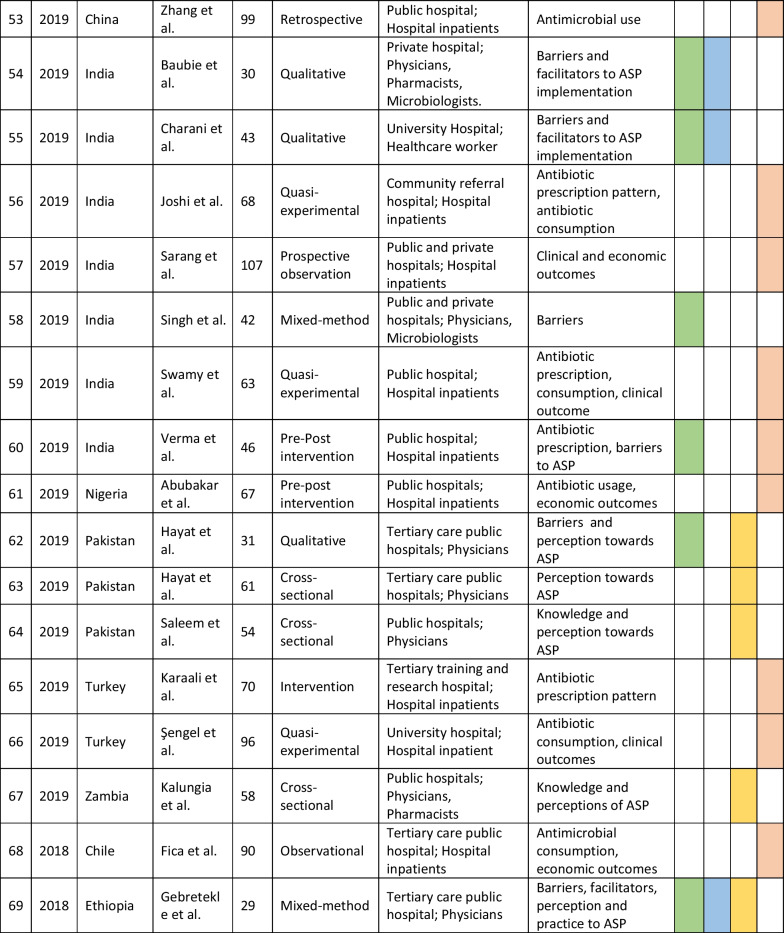

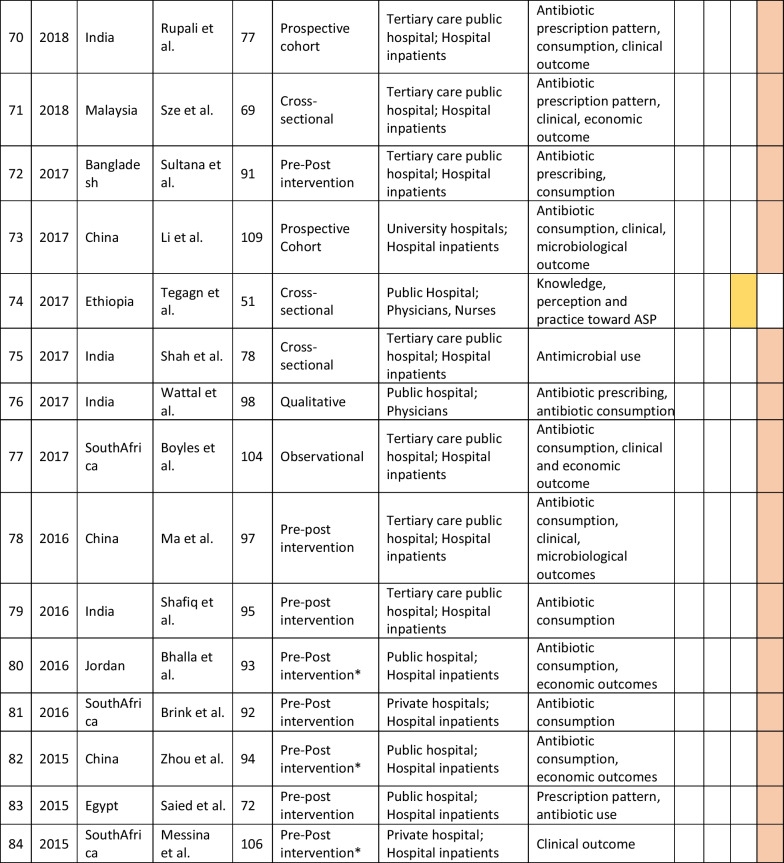
Summary of characteristics of included studies in the scoping review of antimicrobial stewardship programs in low and middle-income countries, 2015–2023

*Assessment by the authors as the study design was not specified in the reference

**Table 2:**
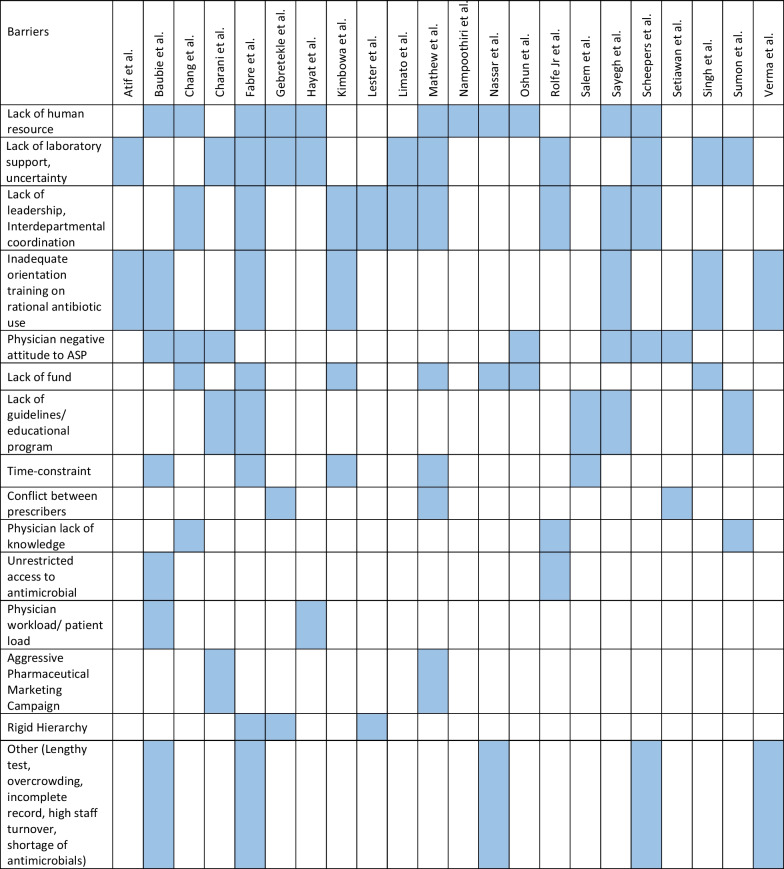
Barriers to implementing ASP of included studies in the scoping review of antimicrobial stewardship programs in low and middle-income countries, 2015–2023

**Table 3:**
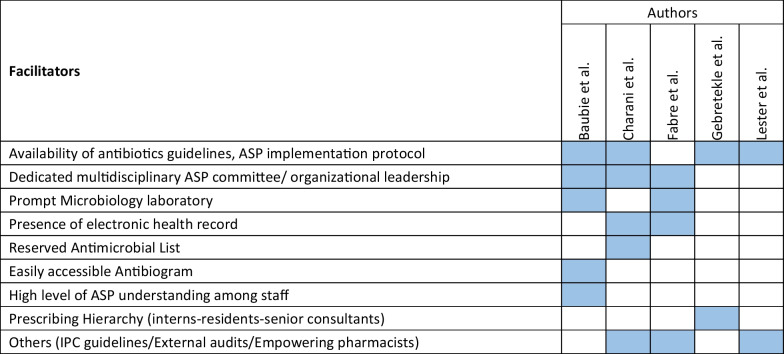
Facilitators for implementing ASP of included studies in the scoping review of antimicrobial stewardship programs in low and middle-income countries, 2015–2023

**Table 4:**
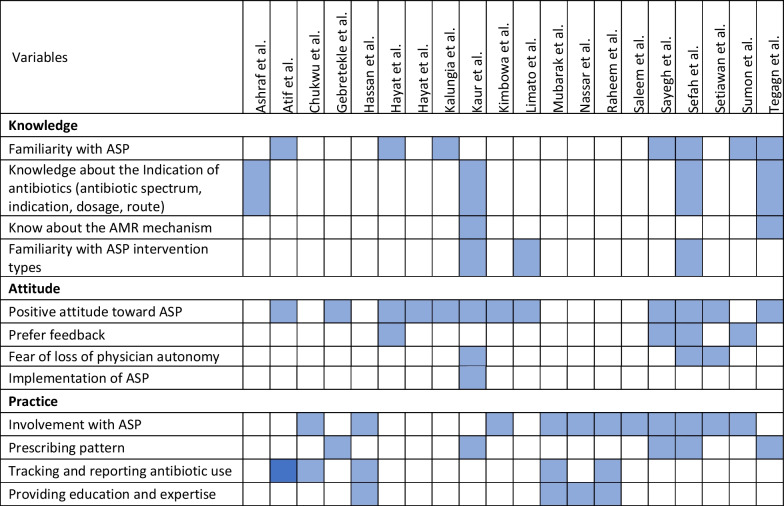
Physicians’ perception and practices on ASP of included studies in the scoping review of antimicrobial stewardship programs in low and middle-income countries, 2015–2023

**Table 5:**
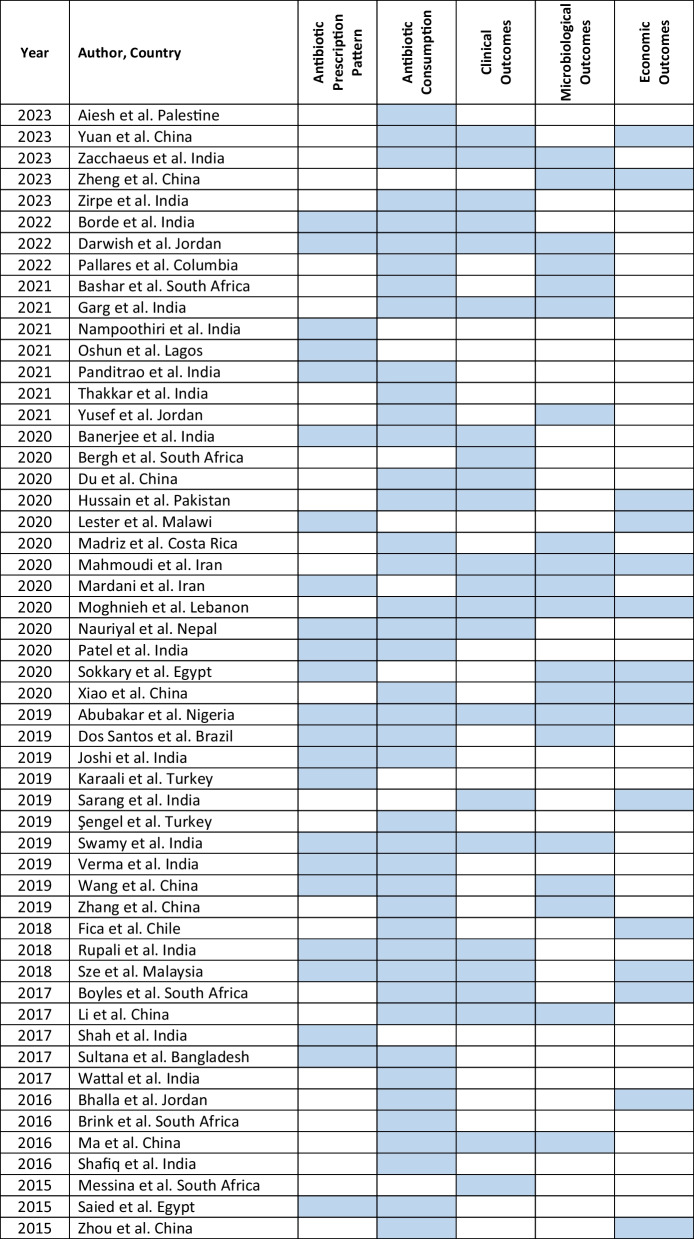
Impact of ASP intervention of included studies in the scoping review of antimicrobial stewardship programs in low and middle-income countries, 2015–2023

## Results

### Characteristics of included studies

**Fig. 1:**
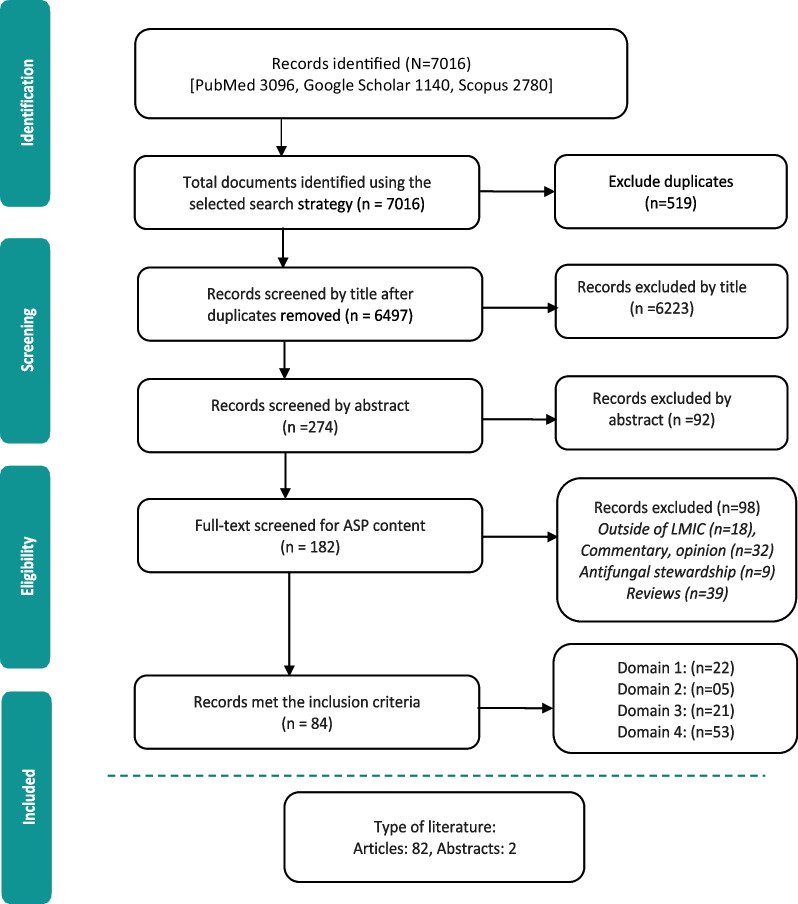
PRISMA flowchart of the selection of studies in the scoping review of antimicrobial stewardship programs in low and middle-income countries, 2015–2023

We initially retrieved 7016 relevant articles through comprehensive and systematic database searches in Pubmed, Google Scholar, and Scopus. After removing duplicates, 6497 records remained for screening. Title and abstract screening resulted in the identification of 182 relevant articles, of which 84 met the inclusion criteria (References 27–110). Ninety-eight articles were excluded based on the exclusion criteria (Fig. 1). The majority of studies (63%, 53/84) reported the impact of ASP interventions, followed by barriers to ASP implementation (26%, 22/84), ASP perceptions and practices of prescribers (25%, 21/84), and facilitators for ASP establishment (6%, 5/84). The studies included in the review were conducted in 34 countries, with India (26%, 22/84), China (12%, 10/84), and Pakistan (10%, 8/84) being the most represented. South Asia had the highest number of articles (40%, 34/84) followed by the Middle East (15%, 13/84) and East Asia (12%, 10/84). The Caribbean region has no representation in our review. In terms of study design, most of the studies were cross-sectional (33%, 28/84), followed by pre-post (14%, 12/84) and quasi-experimental (12%, 10/84). More than half of the studies were single-centered (52%, 44/84) and, 58% (49/84) of the studies were conducted between 2020 and 2023 (Table 1).

### Barriers to implementing ASP

Table 2 summarizes the reported barriers to ASP implementation. Of the 22 studies that documented barriers to ASP adoption, 50% (11/22) reported a shortage of human resources [27–37]. Eleven studies each identified the absence of leadership and minimal governmental support [27, 28, 35–42], and a lack of reliable laboratory infrastructure and microbiology laboratory support as challenges to effective ASP implementation [28, 29, 31, 35, 36, 38, 40, 42–45]. Other identified barriers included inadequate orientation and training on ASP including rational antimicrobial use [27, 30, 36, 41, 44, 46], prescribers’ negative attitudes to changes in antibiotic practices [27, 30, 33, 35, 37, 43, 47], and a lack of dedicated ASP funds [28, 32, 33, 36, 37, 41]. Five studies each cited the absence of approved national guidelines or educational programs [27, 36, 43, 45, 48], and time constraints for prescribers [28, 30, 36, 41, 48] as barriers. The ‘Others’ category included challenges like incomplete electronic medical records (EMR), long waiting times for tests, overcrowding in wards [30, 46], the perception of AMR as an ‘external problem’ [49], frequent staff turnover, lack of specific ASP goals [36], stock shortage of antimicrobials [35], and suboptimal salary [36].

### Facilitators to establishing ASP

The facilitators of hospital ASP implementation are shown in Table 3. Of the five studies documenting ASP enablers, the availability of antibiotic guidelines based on WHO AWaRe classification and ASP protocols was cited as a facilitator for the hospital’s ASP implementation in four studies [29, 30, 39, 43]. The establishment of a dedicated multidisciplinary ASP committee [30, 36, 43] was mentioned as an enabling factor for ASP implementation in three studies. Prompt access to microbiology laboratory facilities with institutional antibiograms [30, 36], the presence of electronic health records [36, 43], and a restricted antimicrobial list in hospitals [43] were also noted as enablers for stewardship implementation. The hospital antibiogram is a periodic summary of antimicrobial susceptibilities of local bacterial isolates which are used by prescribers to determine local susceptibility rates, monitor antibiotic resistance trends over time within the institution, and compare antibiotic resistance trends between hospitals [36]. ‘Others’ facilitating factors included the presence of a guideline for infection prevention and control [39, 43], external audits with feedback, and empowerment of pharmacists [36].

### ASP perception and practices of physicians

Table 4 presents the 21 publications related to physicians’ perceptions and practices (i.e. KAP) regarding ASP. Six of these studies (29%, 6/21) reported on all three elements: knowledge, attitude, and practice [27, 45, 47, 50–52], and six others exclusively assessed ASP practice compliance [32, 53–57]. The majority of the studies revealed that prescribers (physicians or pharmacists) had a sub-optimal knowledge of ASP and its basic principles [31, 45, 47, 51, 52, 58]. However, four studies stated that prescribers had sufficient knowledge of the correct antibiotic indication (i.e. correct dose, diagnosis, and duration of antibiotic) [50–52, 59]. In terms of attitude towards ASP implementation, the majority of prescribers showed positive attitudes, as they felt ASP is beneficial for both prescribers and patients [27, 29, 31, 45, 49, 51, 52, 60, 61], and were interested receive feedback on their antibiotic prescriptions [27, 31, 45, 52]. However, some studies also revealed the physicians’ concern that implementation of ASP would limit their prescribing autonomy [47, 50, 52]. Regarding ASP practice, studies reported that prescribers mostly had a substandard level of involvement in ASP activities or previously worked in ASP facilities [44, 45, 51, 52, 56]. Prescribers also had a low level of compliancein sending specimens for culture and susceptibility tests [44, 45, 52, 60]. However, tracking, reporting, and documenting antibacterial use in patient care had a better level of implementation among prescribers [29, 32, 52, 53, 60].

### Impact of ASP intervention

The majority of studies (63%, 53/84) measured the impact of ASP interventions, which is displayed in five categories in Table 5. The impact of ASP interventions on the appropriateness of antibiotic prescriptions administered was reported in (43%, 23/53) of studies [33, 34, 39, 46, 62–80]. Metrics used to measure appropriate prescriptions included dose adjustment or dose optimization [46, 63, 64], antibiotic de-escalation [62, 63, 68, 74, 80, 81], and timing and duration of antibiotic prophylaxis [67, 70, 76]. Most studies (79%, 42/53) primarily documented a decrease in antibiotic consumption and an improvement in rational antibiotic use after ASP implementation in hospitals [39, 46, 62, 64, 66–69, 71, 73, 76–104]. Metrics for evaluating antibiotic consumption included defined daily doses (DDDs) or days of therapy (DOT) per 1,000 patient-days [62–64, 68, 72, 77, 79–81, 84–86, 88, 100, 104, 105] while some studies used defined daily doses (DDDs) per 100 patient-days or Days of therapy/DOT per 100 patient-days [66, 67, 76, 90, 97, 102].

To assess the impact of ASP interventions, 24 studies (45%, 24/53) assessed clinical or patient-centered outcomes [62–64, 66, 67, 69, 73–75, 77, 79–81, 86–88, 97, 100–104, 106–108]. Metrics used to evaluate the intervention included length of stay in hospital (LOS) in specific units (e.g., ICU) [46, 62, 68, 69, 73, 74, 76, 77, 81, 86, 88, 96, 101–103], hospital mortality [62, 76, 77, 80, 86, 100–102, 106], rehospitalization [77, 81, 83, 86, 104], hospital-acquired infections [63, 64, 67, 81, 97, 107, 109], and device-associated infections [64]. ASPs brought about positive clinical outcomes in hospital inpatients in most cases. Microbiological outcomes to assess the impact of ASP were reported in 42% (22/53) of studies [63, 65, 66, 71, 76, 79, 81–85, 87–89, 100, 101, 109, 110], including a decrease in the prevalence of multi-drug resistance bacterial strains like *Acinetobacter spp*, *Methicillin-Resistant Staphylococcus aureus* (MRSA)*, Vancomycin-Resistant Enterococcus* [VRE], increase in the antibiotic susceptibility of bacterial strains such as *Pseudomonas aeruginosa, E. coli* [71, 81, 82, 85]. Studies also documented a reduction in the incidence of bacterial infections such as Clostridioides difficile infections and Candidemia [75, 77], and a decrease in pan-drug resistant isolates [65, 71, 77, 85]. Economic outcomes were assessed in 17 studies [39, 65, 69, 71, 81, 86, 88–90, 93, 94, 96, 100, 103, 104, 107, 110], and reported a decrease in hospital antibiotic procurement cost [65, 71, 81, 90, 93, 94], savings in antibiotic costs [39, 69, 86, 88, 104, 110], decrease in antibiotic therapy and antibiotic prophylaxis cost [67, 107], and hospitalization costs [110].

## Discussion

The purpose of this scoping review was to look into the present state of hospital-based ASPs in low-and middle-income countries (LMICs), providing insight into four major domains in a stepwise manner: the obstacles, enablers, attitudes, and behaviors of prescribers, and the outcomes of ASP interventions. Our review identified a shortage of human resources, and a lack of diagnostic facilities as the most commonly encountered barriers to ASP implementation, while the availability of hospital ASP guidelines and dedicated multidisciplinary teams were found to be facilitators. Additionally, the review revealed a substandard baseline about ASP perception among physicians and found that ASP interventions were successful in improving the rational use of antibiotics, reducing antibiotic consumption, and decreasing hospital antibiotic procurement costs.

Our review identified several challenges to implementing ASP in resource-compromised settings, including inadequate human resources, a lack of laboratory infrastructure, unreliable institutional antibiogram, the absence of national guidelines, minimal funding, and a lack of ASP orientation and training [27–30, 35–37, 43, 44]. These findings are in line with a previous review, which documented the presence of substandard microbiology laboratory facilities and unreliable antibiograms [1]. Physicians’ lack of trust in microbiology findings poses a serious threat, as prescribers may lean toward broad-spectrum empiric therapy based on anecdotal evidence. Regarding the absence of context-based antibiotic guidelines and lack of ASP training, our findings are reinforced by prior reviews which also documented these factors as impediments [1, 12, 23, 111]. The absence of guidelines implies that prescribers would be unaware of local AMR patterns, leading to the prescription of broad-spectrum antibiotics and the promotion of AMR. Physicians who lack ASP training may administer antibiotics empirically out of habit, and this insufficient training may result in suboptimal ASP understanding among prescribers.

In terms of facilitators toward ASP implementation, existing hospital ASP protocols or guidelines, including their easy access were cited as facilitating factors [29, 30, 39, 43]. The presence of guidelines implies that prescribers have a tool that directs them toward prescribing narrow-spectrum antibiotics. Readily available antibiograms suggest that physicians have a trustworthy source of local, relevant antibiotic patterns, which helps them prescribe antibiotics rationally, instead of resorting to a range of resources.

Studies in our review documented a substandard level of knowledge and familiarity among prescribers with the basic principles of ASP. An inadequate fundamental understanding of ASP and AMR would lead to irrational and improper antimicrobial prescriptions [44]. Regarding perceptions toward ASP, studies reported positive perceptions toward ASP, as physicians felt that ASP made them think more carefully about their antibiotic choices, beneficial in terms of reducing AMR, decreasing patient length of stay, and reducing healthcare costs [27, 31, 49–52, 58]. Our review revealed that prescribers were on board to receive regular monitoring and feedback on rational antibiotic use and recommended tailored ASP training [27, 45, 52, 61]. However, a few studies also revealed that physicians were concerned that ASP implementation might lead to a loss in their prescribing autonomy [27, 47, 52]. Despite the overall favorable perceptions, most countries have yet to align with global efforts to combat increasing AMR through stewardship activities. Studies in this review documented that hospitals either did not have any ASP initiatives in place [24, 45, 50] or had few ASP activities with low compliance levels [44, 53–55]. This low level of ASP compliance might stem from perceiving AMR as an ‘external problem’, with some prescribers having a notion that AMR is developed by inappropriate antibiotic use elsewhere instead of on their premises.

Our review found that ASP interventions achieved improvement in rational antibiotic prescription through dose optimization, antibiotic de-escalation, and a reduction in antibiotic prescriptions. ASP implementation documented a reduction in the consumption of WATCH category antibiotics such as vancomycin, meropenem, aztreonam, ceftriaxone, and RESERVE category antimicrobials like colistin, carbapenems, teicoplanin, in hospital wards and ICUs [46, 63, 71, 77, 82, 83, 100–102]. Prior systematic reviews demonstrated a decrease in antibiotic consumption after ASP implementation in the ICUs of hospitals [2, 24]. This decline in consumption might have been achieved by a combination of factors like revising existing hospital antibiotic policies, compliance with antimicrobial policies, stopping orders at 48 h, de-escalation of empirical antimicrobial therapy, and a curb-on combination therapy [46, 63]. As observed in the review, there was substantial heterogeneity between studies concerning metrics to quantify antibiotic use and consumption. The consumption metrics used were DDD with different denominators (100 or 1000 patient-days) or Days of therapy/DOT per 100 patient-days or 1000 patient-days. This lack of uniformity makes it difficult to compare, aggregate, and interpret data. While the use of different metrics makes it difficult for an accurate and meaningful comparison, an International multidisciplinary panel recommended the simultaneous use of at least two metrics to quantify antibiotic use in hospital settings. DDDs per 100(0) patient-days and days of therapy per patient-days were identified as the most common metric used in the hospital setting [112]. Clinical outcomes such as a decrease in the length of hospital stay (LOS), in-hospital mortality, and a reduction in Clostridioides difficile infections were observed after ASP implementation [46, 62, 63, 69, 81, 86, 102]. Prior systematic reviews also confirmed that stewardship activities resulted in positive clinical outcomes [19, 113]. These findings imply that ASPs curb needless antibiotic consumption among hospital inpatients, and show a positive impact on microbiological outcomes, such as a reduction in the prevalence of multi-drug resistance [82, 83, 88] and bacterial resistance [82, 83]. Though fewer studies reported ASPs’ impact on economic outcomes, the studies in this review showed a positive effect on ASP implementation which was reiterated by prior reviews that revealed the beneficial effects of ASPs in terms of cost reduction in clinical settings [15, 114]. ASPs were able to reduce the needless use of expensive parenteral antibiotics and demonstrated a decrease in the use of high-cost broad-spectrum antibiotics [69, 88]. These pieces of evidence show that ASPs were successful in accomplishing the core objective of reducing inappropriate antibiotic use and antibiotic costs without compromising clinical outcomes.

Clinical outcomes are essential objectives of ASPs that justify the long-term sustainability of any ASP program. Most of the studies included in our review were unable to meet this expectation. Future ASP initiatives should attempt to include and report microbiological, clinical, and cost-effectiveness outcomes. In this review, barring only four studies [77, 103, 108, 109], none of the other citations had a control group. Owing to the lack of a control group and the non-randomized design of most of the studies, confounding effects would be difficult to control. Since hospital settings can also differ across LMICs, this might also result in confounders affecting the prescribing pattern and patient-centered outcomes. If randomization is difficult to conduct, future ASP attempts should at least try to include a control group to minimize confounding and make the results more definitive. The majority of the studies in this scoping review were single-centered, with almost all of them being conducted in tertiary care centers of urban settings. This limits the generalizability of the review since our findings are in line with a recent review that also found that the majority of ASP studies were conducted in urban areas. [24]. Implementation of hospital ASP in rural settings of LMICs might also be more challenging owing to financial and resource constraints. Future efforts should emphasize conducting multi-center trials and if possible in rural settings. Studies in this review had a short follow-up period ranging from 2 to 6 months [46, 65, 67, 69, 72, 77, 86, 102], with some studies having no defined follow-up period [64, 91]. Shortage of the follow-up period would make it difficult to accurately assess microbiological outcomes such as changes in AMR pattern, hospital re-admission, mortality, and determination of the long-term impact of ASP on antimicrobial cost reductions. Long-term evidence of positive economic outcomes would also help to convince stakeholders and policymakers to invest in the ASP program. Furthermore, the implementation of infection prevention and control (IPC) programs to prevent bacterial infections and infections will be necessary to improve patient safety and healthcare quality [14]. This approach is strongly linked to ASP, as co-implemented with IPC measures were successful in curbing AMR [115], and future ASP interventions can integrate basic IPC measures into ASP programs. To implement ASP in a resource-compromised hospital setting, WHO recommends the establishment of a multidisciplinary ASP team, comprising infectious diseases specialists, microbiologists, nurses with IPC expertise, and clinical pharmacists as core members [116]. Of the 53 studies reporting stewardship interventions, only five studies documented the involvement of multidisciplinary teams with such composition [39, 82, 86, 92, 105]. Future ASP efforts should concentrate on forming a more inclusive multidisciplinary team to ensure the long-term sustainability of stewardship activities.

Our scoping review sequentially integrates the four ASP domains of barriers, facilitators, prescriber perceptions and practices, and intervention impact to provide a comprehensive overview of the current state of ASP in LMICs, representing cumulative evidence from almost all LMIC regions except the Caribbean. However, this review has a few limitations. We only included articles published in English due to linguistic constraints, which may have resulted in the exclusion of relevant studies in other languages. Albeit the aforementioned limitation, Indian and Pakistani citations are mostly published in English. In addition, our review included global representation except the Carribean. Additionally, the scope of our review was limited to studies published between 2015 and 2023 and there may be earlier studies that could have provided significant insights. However, since hospital-based ASP articles in LMICs increased following the WHO GAP Plan on AMR in 2015, and we included publication from 2015 onwards, along with the most recent citations, this drawback is negated to a substantial extent. Finally, we did not analyze the possibility of bias for the selected research. We excluded documents such as case studies, commentaries, and expert opinions which have a high potential for bias with weak evidence.

## Conclusions

Antimicrobial stewardship programs in LMICs face a range of challenges, including a shortage of human resources, inadequate laboratory infrastructure, limited governmental support, and a lack of national guidelines. However, the availability of hospital ASP guidelines and protocols is a significant facilitator for ASP implementation. Physicians in LMICs generally have a suboptimal level of knowledge and familiarity with antimicrobial stewardship programs, but they hold positive attitudes toward ASP and are willing to receive training and educational sessions. ASP interventions in LMICs have been effective in improving the rational use of antibiotics, leading to reduced antibiotic consumption, improved clinical outcomes, positive microbiological outcomes, and economic benefits. Future efforts should focus on addressing the identified barriers to ASP implementation, involving multidisciplinary teams in stewardship initiatives, and integrating infection prevention and control measures into ASP programs to combat antimicrobial resistance effectively in LMICs.

### Supplementary Information

**Additional file 1: Appendix 1**. Search strategy.

## Data Availability

The datasets used and/or analyzed during the current study are available from the corresponding author upon reasonable request.

## Abbreviations

ASP: Antimicrobial stewardship program
AMR: Antimicrobial resistance
GAP: Global Action Plan
DOT: Days of therapy
DDDs: Defined daily doses
GDP: Gross Domestic Product
IPC: Infection prevention and control
ICU: Intensive Care Unit
KAP: Knowledge, attitude, and practices
LOS: Length of stay in hospital
LMICs: Low-and-middle-income countries
MeSH: Medical subject headings
PRISMA-ScR: Preferred Reporting Items for Systematic Reviews and Meta-Analysis extension for scoping reviews
WHO: World Health Organization

## References

[1] Kakkar AK, Shafiq N, Singh G, Ray P, Gautam V, Agarwal R, Muralidharan J, Arora P (2020). Antimicrobial stewardship programs in resource constrained environments: understanding and addressing the need of the systems. Front Public Health.

[2] Van Dijck C, Vlieghe E, Cox JA (2018). Antibiotic stewardship interventions in hospitals in low-and middle-income countries: a systematic review. Bull World Health Organ.

[3] Murray CJ, Ikuta KS, Sharara F, Swetschinski L, Aguilar GR, Gray A, Han C, Bisignano C, Rao P, Wool E, Johnson SC (2022). Global burden of bacterial antimicrobial resistance in 2019: a systematic analysis. Lancet.

[4] Mestrovic T, Aguilar GR, Swetschinski LR, Ikuta KS, Gray AP, Weaver ND, Han C, Wool EE, Hayoon AG, Hay SI, Dolecek C (2022). The burden of bacterial antimicrobial resistance in the WHO European region in 2019: a cross-country systematic analysis. Lancet Public Health.

[5] Harun MGD, Anwar MMU, Sumon SA, Hassan MZ, Haque T, Mah EMS, Rahman A, Abdullah S, Islam MS, Styczynski AR, Kaydos-Daniels SC (2022). Infection prevention and control in tertiary care hospitals of Bangladesh: results from WHO infection prevention and control assessment framework (IPCAF). Antimicrob Resist Infect Control.

[6] Pokharel S, Raut S, Adhikari B (2019). Tackling antimicrobial resistance in low-income and middle-income countries. BMJ Glob Health.

[7] Mzumara GW, Mambiya M, Iroh Tam PY (2021). Antimicrobial stewardship interventions in least developed and low-income countries: a systematic review protocol. BMJ Open.

[8] Morgan DJ, Okeke IN, Laxminarayan R, Perencevich EN, Weisenberg S (2011). Non-prescription antimicrobial use worldwide: a systematic review. Lancet Infect Dis.

[9] Zellweger RM, Carrique-Mas J, Limmathurotsakul D, Day NPJ, Thwaites GE, Baker S (2017). A current perspective on antimicrobial resistance in Southeast Asia. J Antimicrob Chemother.

[10] Akpan MR, Isemin NU, Udoh AE, Ashiru-Oredope D (2020). Implementation of antimicrobial stewardship programmes in African countries: a systematic literature review. J Glob Antimicrob Resist.

[11] Chetty S, Reddy M, Ramsamy Y, Naidoo A, Essack S (2019). Antimicrobial stewardship in South Africa: a scoping review of the published literature. JAC Antimicrob Resist.

[12] Laxminarayan R, Duse A, Wattal C, Zaidi AK, Wertheim HF, Sumpradit N, Vlieghe E, Hara GL, Gould IM, Goossens H (2013). Antibiotic resistance-the need for global solutions. Lancet Infect Dis.

[13] Mendelson M, Matsoso MP (2015). The World Health Organization Global Action Plan for antimicrobial resistance. S Afr Med J.

[14] Harun MGD, Anwar MMU, Sumon SA, Hassan MZ, Mohona TM, Rahman A, Abdullah S, Islam MS, Kaydos-Daniels SC, Styczynski AR (2022). Rationale and guidance for strengthening infection prevention and control measures and antimicrobial stewardship programs in Bangladesh: a study protocol. BMC Health Serv Res.

[15] Nathwani D, Varghese D, Stephens J, Ansari W, Martin S, Charbonneau C (2019). Value of hospital antimicrobial stewardship programs [ASPs]: a systematic review. Antimicrob Resist Infect Control.

[16] Hegewisch-Taylor J, Dreser-Mansilla A, Romero-Mónico J, Levy-Hara G (2020). Antimicrobial stewardship in hospitals in Latin America and the Caribbean: a scoping review. Rev Panam Salud Publica.

[17] Kaki R, Elligsen M, Walker S, Simor A, Palmay L, Daneman N (2011). Impact of antimicrobial stewardship in critical care: a systematic review. J Antimicrob Chemother.

[18] Septimus EJ, Owens RC (2011). Need and potential of antimicrobial stewardship in community hospitals. Clin Infect Dis.

[19] Schuts EC, Hulscher M, Mouton JW, Verduin CM, Stuart J, Overdiek H, van der Linden PD, Natsch S, Hertogh C, Wolfs TFW (2016). Current evidence on hospital antimicrobial stewardship objectives: a systematic review and meta-analysis. Lancet Infect Dis.

[20] Davey P, Brown E, Charani E, Fenelon L, Gould IM, Holmes A, Ramsay CR, Wiffen PJ, Wilcox M (2013). Interventions to improve antibiotic prescribing practices for hospital inpatients. Cochrane Database Syst Rev.

[21] Hulscher M, Prins JM (2017). Antibiotic stewardship: does it work in hospital practice? A review of the evidence base. Clin Microbiol Infect.

[22] Mendelson M, Røttingen JA, Gopinathan U, Hamer DH, Wertheim H, Basnyat B, Butler C, Tomson G, Balasegaram M (2016). Maximising access to achieve appropriate human antimicrobial use in low-income and middle-income countries. Lancet.

[23] Cox JA, Vlieghe E, Mendelson M, Wertheim H, Ndegwa L, Villegas MV, Gould I, Levy Hara G (2017). Antibiotic stewardship in low- and middle-income countries: the same but different?. Clin Microbiol Infect.

[24] Setiawan E, Abdul-Aziz MH, Roberts JA, Cotta MO (2022). Hospital-based antimicrobial stewardship programs used in low- and middle-income countries: a scoping review. Microb Drug Resist.

[25] Tricco AC, Lillie E, Zarin W, O'Brien KK, Colquhoun H, Levac D, Moher D, Peters MDJ, Horsley T, Weeks L (2018). PRISMA extension for scoping reviews (PRISMA-ScR): checklist and explanation. Ann Intern Med.

[26] Bank W (2020). World bank data.

[27] Sayegh N, Hallit S, Hallit R, Saleh N, Zeidan RK (2021). Physicians' attitudes on the implementation of an antimicrobial stewardship program in Lebanese hospitals. Pharm Pract (Granada).

[28] Mathew P, Ranjalkar J, Chandy SJ (2020). Challenges in implementing antimicrobial stewardship programmes at secondary level hospitals in India: an exploratory study. Front Public Health.

[29] Gebretekle GB, Haile Mariam D, Abebe W, Amogne W, Tenna A, Fenta TG, Libman M, Yansouni CP, Semret M (2018). Opportunities and barriers to implementing antibiotic stewardship in low and middle-income countries: lessons from a mixed-methods study in a tertiary care hospital in Ethiopia. PLoS ONE.

[30] Baubie K, Shaughnessy C, Kostiuk L, Varsha Joseph M, Safdar N, Singh SK, Siraj D, Sethi A, Keating J (2019). Evaluating antibiotic stewardship in a tertiary care hospital in Kerala, India: a qualitative interview study. BMJ Open.

[31] Hayat K, Rosenthal M, Gillani AH, Zhai P, Aziz MM, Ji W, Chang J, Hu H, Fang Y (2019). Perspective of Pakistani physicians towards hospital antimicrobial stewardship programs: a multisite exploratory qualitative study. Int J Environ Res Public Health.

[32] Nassar H, Abu-Farha R, Barakat M, Alefishat E (2022). Antimicrobial stewardship from health professionals' perspective: awareness, barriers, and level of implementation of the program. Antibiotics (Basel).

[33] Oshun P, Roberts A, Osuagwu C, Akintan P, Fajolu I, Ola-Bello O, Odukoya O, Akodu B, Okunowo A, Versporten A (2021). Roll out of a successful antimicrobial stewardship programme in Lagos university teaching hospital Nigeria using the Global-Point prevalence survey. Afr J Clin Exp Microbiol.

[34] Nampoothiri V, Sudhir AS, Joseph MV, Mohamed Z, Menon V, Charani E, Singh S (2021). Mapping the implementation of a clinical pharmacist-driven antimicrobial stewardship programme at a tertiary care centre in South India. Antibiotics (Basel).

[35] Scheepers LN, Niesing CM, Bester P (2023). Facilitators and barriers to implementing antimicrobial stewardship programs in public South African hospitals. Antimicrob Steward Healthc Epidemiol.

[36] Fabre V, Secaira C, Cosgrove SE, Lessa FC, Patel TS, Alvarez AA, Anchiraico LM, Del Carmen BM, Barberis MF, Burokas MS (2023). Deep dive into gaps and barriers to implementation of antimicrobial stewardship programs in hospitals in Latin America. Clin Infect Dis.

[37] Chang FY, Chuang YC, Veeraraghavan B, Apisarnthanarak A, Tayzon MF, Kwa AL, Chiu CH, Deris ZZ, Amir Husin S, Hashim H (2022). Gaps in antimicrobial stewardship programmes in Asia: a survey of 10 countries. JAC Antimicrob Resist.

[38] Rolfe R, Kwobah C, Muro F, Ruwanpathirana A, Lyamuya F, Bodinayake C, Nagahawatte A, Piyasiri B, Sheng T, Bollinger J (2021). Barriers to implementing antimicrobial stewardship programs in three low- and middle-income country tertiary care settings: findings from a multi-site qualitative study. Antimicrob Resist Infect Control.

[39] Lester R, Haigh K, Wood A, MacPherson EE, Maheswaran H, Bogue P, Hanger S, Kalizang'oma A, Srirathan V, Kulapani D (2020). Sustained reduction in third-generation cephalosporin usage in adult inpatients following introduction of an antimicrobial stewardship program in a large, urban hospital in Malawi. Clin Infect Dis.

[40] Limato R, Broom A, Nelwan EJ, Hamers RL (2022). A qualitative study of barriers to antimicrobial stewardship in Indonesian hospitals: governance, competing interests, cost, and structural vulnerability. Antimicrob Resist Infect Control.

[41] Kimbowa IM, Ocan M, Eriksen J, Nakafeero M, Obua C, Stålsby Lundborg C, Kalyango J (2022). Characteristics of antimicrobial stewardship programmes in hospitals of Uganda. PLoS ONE.

[42] Singh S, Charani E, Wattal C, Arora A, Jenkins A, Nathwani D (2019). The state of education and training for antimicrobial stewardship programs in indian hospitals: a qualitative and quantitative assessment. Antibiotics (Basel).

[43] Charani E, Smith I, Skodvin B, Perozziello A, Lucet JC, Lescure FX, Birgand G, Poda A, Ahmad R, Singh S, Holmes AH (2019). Investigating the cultural and contextual determinants of antimicrobial stewardship programmes across low-, middle- and high-income countries: a qualitative study. PLoS ONE.

[44] Atif M, Ihsan B, Malik I, Ahmad N, Saleem Z, Sehar A, Babar ZU (2021). Antibiotic stewardship program in Pakistan: a multicenter qualitative study exploring medical doctors' knowledge, perception and practices. BMC Infect Dis.

[45] Sumon SA, Islam S, Harun GD (2022). Perceptions toward and practices regarding antibiotic stewardship and use among physicians at tertiary-care public hospitals in Bangladesh. Antimicrob Steward Healthc Epidemiol.

[46] Verma M, Shafiq N, Tripathy JP, Nagaraja SB, Kathirvel S, Chouhan DK, Arora P, Singh T, Jain K, Gautam V, Dhillon MS (2019). Antimicrobial stewardship programme in a trauma centre of a tertiary care hospital in North India: effects and implementation challenges. J Glob Antimicrob Resist.

[47] Setiawan E, Cotta MO, Abdul-Aziz MH, Sosilya H, Widjanarko D, Wardhani DK, Roberts JA (2022). Indonesian healthcare providers' perceptions and attitude on antimicrobial resistance, prescription and stewardship programs. Future Microbiol.

[48] Salem MR, Youssef MRL, Shalaby SF, Mahmoud AT, Ismail M, Ibrahim SK (2023). Perspectives on antibiotic stewardship programs among health care providers at two university hospitals in Egypt. Int J Environ Res Public Health.

[49] Limato R, Nelwan EJ, Mudia M, Alamanda M, Manurung ER, Mauleti IY, Mayasari M, Firmansyah I, Djaafar R, Vu HTL (2022). Perceptions, views and practices regarding antibiotic prescribing and stewardship among hospital physicians in Jakarta, Indonesia: a questionnaire-based survey. BMJ Open.

[50] Kaur S, Sethi P, Panda PK. Knowledge-practice gaps of practicing doctors on antimicrobial stewardship: a single center experience. 2022.

[51] Tegagn G, Yadesa T, Ahmed Y (2017). Knowledge, attitudes and practices of healthcare professionals towards antimicrobial stewardship and their predictors in Fitche Hospital. J Bioanal Biomed.

[52] Sefah IA, Chetty S, Yamoah P, Meyer JC, Chigome A, Godman B, Bangalee V (2023). A multicenter cross-sectional survey of knowledge, attitude, and practices of healthcare professionals towards antimicrobial stewardship in Ghana: findings and implications. Antibiotics.

[53] Mubarak N, Khan AS, Zahid T, Ijaz UEB, Aziz MM, Khan R, Mahmood K, Saif-Ur-Rehman N, Zin CS (2021). Assessment of adherence to the core elements of hospital antibiotic stewardship programs: a survey of the Tertiary Care Hospitals in Punjab, Pakistan. Antibiotics (Basel).

[54] Saleem Z, Hassali MA, Hashmi FK, Godman B, Ahmed Z (2019). Snapshot of antimicrobial stewardship programs in the hospitals of Pakistan: findings and implications. Heliyon.

[55] Raheem M, Anwaar S, Aziz Z, Raja SA, Saif-Ur-Rehman N, Mubarak N (2020). Adherence to the core elements of outpatient antibiotic stewardship: a cross-sectional survey in the tertiary care hospitals of Punjab, Pakistan. Infect Drug Resist.

[56] Chukwu EE, Oshun PO, Osuolale KA, Chuka-Ebene VO, Salako A, Idigbe IE, Oladele D, Audu RA, Ogunsola FT (2021). Antimicrobial stewardship programmes in healthcare facilities in Lagos State, Nigeria: a needs assessment. J Glob Antimicrob Resist.

[57] Hassan SK, Dahmash EZ, Madi T, Tarawneh O, Jomhawi T, Alkhob W, Ghanem R, Halasa Z (2023). Four years after the implementation of antimicrobial stewardship program in Jordan: evaluation of program's core elements. Front Public Health.

[58] Kalungia AC, Mwambula H, Munkombwe D, Marshall S, Schellack N, May C, Jones ASC, Godman B (2019). Antimicrobial stewardship knowledge and perception among physicians and pharmacists at leading tertiary teaching hospitals in Zambia: implications for future policy and practice. J Chemother.

[59] Ashraf S, Ashraf S, Ashraf M, Imran MA, Choudhary ZA, Hafsa HT, Awais AB, Kalsoom L, Farooq I, Habib Z (2022). Knowledge, attitude, and practice of clinicians about antimicrobial stewardship and resistance among hospitals of Pakistan: a multicenter cross-sectional study. Environ Sci Pollut Res Int.

[60] Kimbowa IM, Eriksen J, Nakafeero M, Obua C, Lundborg CS, Kalyango J, Ocan M (2022). Antimicrobial stewardship: attitudes and practices of healthcare providers in selected health facilities in Uganda. PLoS ONE.

[61] Hayat K, Rosenthal M, Zhu S, Gillani AH, Chang J, Bogale AA, Kabba JA, Yang C, Jiang M, Zhao M, Fang Y (2019). Attitude of clinicians towards hospital-based antimicrobial stewardship programs: a multicenter cross-sectional study from Punjab, Pakistan. Expert Rev Anti Infect Ther.

[62] Nauriyal V, Rai SM, Joshi RD, Thapa BB, Kaljee L, Prentiss T, Maki G, Shrestha B, Bajracharya DC, Karki K (2020). Evaluation of an antimicrobial stewardship program for wound and burn care in three hospitals in Nepal. Antibiotics (Basel).

[63] Swamy A, Sood R, Kapil A, Vikram NK, Ranjan P, Jadon RS, Soneja M, Sreenivas V (2019). Antibiotic stewardship initiative in a Medicine unit of a tertiary care teaching hospital in India: a pilot study. Indian J Med Res.

[64] Panditrao A, Shafiq N, Kumar MP, Sekhon AK, Biswal M, Singh G, Kaur K, Ray P, Malhotra S, Gautam V (2021). Impact of an antimicrobial stewardship and monitoring of infection control bundle in a surgical intensive care unit of a tertiary-care hospital in India. J Glob Antimicrob Resist.

[65] El-Sokkary RH, Negm EM, Othman HA, Tawfeek MM, Metwally WS (2020). Stewardship actions for device associated infections: an intervention study in the emergency intensive care unit. J Infect Public Health.

[66] Banerjee S, Gupta N, Ray Y, Kodan P, Khot WY, Fazal F, Nyas VKM, Soneja M, Vikram N, Biswas A (2020). Impact of trainee-driven Antimicrobial Stewardship Program in a high burden resource-limited setting. Infez Med.

[67] Abubakar U, Syed Sulaiman SA, Adesiyun AG (2019). Impact of pharmacist-led antibiotic stewardship interventions on compliance with surgical antibiotic prophylaxis in obstetric and gynecologic surgeries in Nigeria. PLoS ONE.

[68] Joshi RD, Zervos M, Kaljee LM, Shrestha B, Maki G, Prentiss T, Bajracharya D, Karki K, Joshi N, Rai SM (2019). Evaluation of a hospital-based post-prescription review and feedback pilot in Kathmandu, Nepal. Am J Trop Med Hyg.

[69] Sze WT, Kong MC (2018). Impact of printed antimicrobial stewardship recommendations on early intravenous to oral antibiotics switch practice in district hospitals. Pharm Pract (Granada).

[70] Karaali C, Emiroglu M, Atalay S, Sert I, Dursun A, Kose S, Akbulut G, Aydın C (2019). A new antibiotic stewardship program approach is effective on inappropriate surgical prophylaxis and discharge prescription. J Infect Dev Ctries.

[71] Dos Santos RP, Dalmora CH, Lukasewicz SA, Carvalho O, Deutschendorf C, Lima R, Leitzke T, Correa NC, Gambetta MV (2019). Antimicrobial stewardship through telemedicine and its impact on multi-drug resistance. J Telemed Telecare.

[72] Saied T, Hafez SF, Kandeel A, El-kholy A, Ismail G, Aboushady M, Attia E, Hassaan A, Abdel-Atty O, Elfekky E (2015). Antimicrobial stewardship to optimize the use of antimicrobials for surgical prophylaxis in Egypt: a multicenter pilot intervention study. Am J Infect Control.

[73] Du Y, Li J, Wang X, Peng X, Wang X, He W, Li Y, Wang X, Yang Q, Zhang X (2020). Impact of a multifaceted pharmacist-led intervention on antimicrobial stewardship in a gastroenterology ward: a segmented regression analysis. Front Pharmacol.

[74] Patel U, Joshi AS, Ganjiwale J, Ganguly B (2020). Impact of antimicrobial stewardship program on prescribing pattern of cephalosporins in the department of surgery. Natl J Physiol Pharm Pharmacol.

[75] Mardani M, Abolghasemi S, Shabani S (2020). Impact of an antimicrobial stewardship program in the antimicrobial-resistant and prevalence of clostridioides difficile infection and amount of antimicrobial consumed in cancer patients. BMC Res Notes.

[76] Wang H, Wang H, Yu X, Zhou H, Li B, Chen G, Ye Z, Wang Y, Cui X, Zheng Y (2019). Impact of antimicrobial stewardship managed by clinical pharmacists on antibiotic use and drug resistance in a Chinese hospital, 2010–2016: a retrospective observational study. BMJ Open.

[77] Rupali P, Palanikumar P, Shanthamurthy D, Peter JV, Kandasamy S, Zacchaeus NGP, Alexander H, Thangavelu P, Karthik R, Abraham OC (2019). Impact of an antimicrobial stewardship intervention in India: evaluation of post-prescription review and feedback as a method of promoting optimal antimicrobial use in the intensive care units of a tertiary-care hospital. Infect Control Hosp Epidemiol.

[78] Shah N, Joshi A, Ganguly B (2017). Impact of antibiotic stewardship program on prescribing pattern of antimicrobials in patients of medical intensive care unit. J Clin Diagn Res.

[79] Darwish RM, Matar SG, Snaineh AAA, Alsharif MR, Yahia AB, Mustafa HN, Hasabo EA (2022). Impact of antimicrobial stewardship on antibiogram, consumption and incidence of multi drug resistance. BMC Infect Dis.

[80] Borde K, Medisetty MK, Muppala BS, Reddy AB, Nosina S, Dass MS, Prashanthi A, Billuri P, Mathai D (2022). Impact of an antimicrobial stewardship intervention on usage of antibiotics in Coronavirus disease-2019 at a Tertiary Care Teaching Hospital in India. IJID Reg.

[81] Moghnieh R, Awad L, Abdallah D, Jadayel M, Sinno L, Tamim H, Jisr T, El-Hassan S, Lakkis R, Dabbagh R, Bizri AR (2020). Effect of a “handshake” stewardship program versus a formulary restriction policy on High-End antibiotic use, expenditure, antibiotic resistance, and patient outcome. J Chemother.

[82] Pallares C, Hernández-Gómez C, Appel TM, Escandón K, Reyes S, Salcedo S, Matta L, Martínez E, Cobo S, Mora L (2022). Impact of antimicrobial stewardship programs on antibiotic consumption and antimicrobial resistance in four Colombian healthcare institutions. BMC Infect Dis.

[83] Yusef D, Hayajneh WA, Bani Issa A, Haddad R, Al-Azzam S, Lattyak EA, Lattyak WJ, Gould I, Conway BR, Bond S (2021). Impact of an antimicrobial stewardship programme on reducing broad-spectrum antibiotic use and its effect on carbapenem-resistant Acinetobacter baumannii (CRAb) in hospitals in Jordan. J Antimicrob Chemother.

[84] Bashar MA, Miot J, Shoul E, van Zyl RL (2021). Impact of an antibiotic stewardship programme in a surgical setting. S Afr J Infect Dis.

[85] Díaz-Madriz JP, Cordero-García E, Chaverri-Fernández JM, Zavaleta-Monestel E, Murillo-Cubero J, Piedra-Navarro H, Hernández-Guillén M, Jiménez-Méndez T (2020). Impact of a pharmacist-driven antimicrobial stewardship program in a private hospital in Costa Rica. Rev Panam Salud Pública.

[86] Hussain K, Khan MF, Ambreen G, Raza SS, Irfan S, Habib K, Zafar H (2020). An antibiotic stewardship program in a surgical ICU of a resource-limited country: financial impact with improved clinical outcomes. J Pharm Policy Pract.

[87] Garg R, Singh G, Kumar S, Verma M, Podder L, Ingle V, Singhai A, Karuna T, Saigal S, Walia K, Khadanga S (2021). Impact of an anti-microbial stewardship program on targeted antimicrobial therapy in a Tertiary Care Health Care Institute in Central India. Cureus.

[88] Mahmoudi L, Sepasian A, Firouzabadi D, Akbari A (2020). The impact of an antibiotic stewardship program on the consumption of specific antimicrobials and their cost burden: a hospital-wide intervention. Risk Manag Healthc Policy.

[89] Xiao Y, Shen P, Zheng B, Zhou K, Luo Q, Li L (2020). Change in antibiotic use in secondary and tertiary hospitals nationwide after a national antimicrobial stewardship campaign was launched in China, 2011–2016: an observational study. J Infect Dis.

[90] Fica A, Valenzuela C, Leiva I, Vergara T, Soto A, Dabanch J, Magunacelaya P (2018). Long-term impact of competitive biddings and an antimicrobial stewardship program in a general hospital in Chile. Rev Med Chil.

[91] Sultana SP, Rahman MS (2017). Dynamic online antimicrobial guideline with stewardship program: impact on antimicrobial prescribing. Bangladesh J Pharmacol.

[92] Brink AJ, Messina AP, Feldman C, Richards GA, Becker PJ, Goff DA, Bauer KA, Nathwani D, van den Bergh D (2016). Antimicrobial stewardship across 47 South African hospitals: an implementation study. Lancet Infect Dis.

[93] Bhalla N, Hussein N, Atari M, Fakhri RM, Lepora C, Walsh N, Cosgrove SE, Murphy RA (2016). Introducing an antibiotic stewardship program in a humanitarian surgical hospital. Am J Infect Control.

[94] Zhou Y, Ma LY, Zhao X, Tian SH, Sun LY, Cui YM (2015). Impact of pharmacist intervention on antibiotic use and prophylactic antibiotic use in urology clean operations. J Clin Pharm Ther.

[95] Shafiq N, Praveen Kumar M, Gautam V, Negi H, Roat R, Malhotra S, Ray P, Agarwal R, Bhalla A, Sharma N (2016). Antibiotic stewardship in a tertiary care hospital of a developing country: establishment of a system and its application in a unit-GASP initiative. Infection.

[96] Şengel BE, Bilgin H, Bilgin BÖ, Gidener T, Saydam S, Pekmezci A, Ergönül Ö, Korten V (2019). The need for an antibiotic stewardship program in a hospital using a computerized pre-authorization system. Int J Infect Dis.

[97] Ma X, Xie J, Yang Y, Guo F, Gao Z, Shao H, Huang Y, Yang C, Qiu H (2016). Antimicrobial stewardship of Chinese ministry of health reduces multidrug-resistant organism isolates in critically ill patients: a pre-post study from a single center. BMC Infect Dis.

[98] Wattal C, Khanna S, Goel N, Oberoi JK, Rao BK (2017). Antimicrobial prescribing patterns of surgical speciality in a tertiary care hospital in India: role of persuasive intervention for changing antibiotic prescription behaviour. Indian J Med Microbiol.

[99] Zhang D, Cui K, Lu W, Bai H, Zhai Y, Hu S, Li H, Dong H, Feng W, Dong Y (2019). Evaluation of carbapenem use in a tertiary hospital: antimicrobial stewardship urgently needed. Antimicrob Resist Infect Control.

[100] Aiesh BM, Nazzal MA, Abdelhaq AI, Abutaha SA, Zyoud SH, Sabateen A (2023). Impact of an antibiotic stewardship program on antibiotic utilization, bacterial susceptibilities, and cost of antibiotics. Sci Rep.

[101] Zacchaeus NGP, Palanikumar P, Alexander H, Webster J, Nair IK, Sadanshiv M, Thomas RM, Deodhar D, Samuel P, Rupali P (2023). Establishing an effective antimicrobial stewardship program at four secondary-care hospitals in India using a hub-and-spoke model. Antimicrob Steward Healthc Epidemiol.

[102] Zirpe K, Kapse US, Gurav SK, Tiwari AM, Deshmukh AM, Suryawanshi PB, Bhoyar AP, Wankhede PP, Desai D, Suryawanshi R (2023). Impact of an antimicrobial stewardship program on broad spectrum antibiotics consumption in the intensive care setting. Indian J Crit Care Med.

[103] Yuan X, Chen K, Yuan J, Chu Q, Hu S, Gao Y, Yu F, Diao X, Chen X, Li Y (2023). Evaluation of the effectiveness and safety of a multi-faceted computerized antimicrobial stewardship intervention in surgical settings: a single-centre cluster-randomized controlled trial. Int J Antimicrob Agents.

[104] Boyles TH, Naicker V, Rawoot N, Raubenheimer PJ, Eick B, Mendelson M (2017). Sustained reduction in antibiotic consumption in a South African public sector hospital; four year outcomes from the Groote Schuur Hospital antibiotic stewardship program. S Afr Med J.

[105] Thakkar P, Singhal T, Shah S, Bhavsar R, Ladi S, John RE, Chavan R, Naik R (2021). The implementation and outcome of a 2-year prospective audit and feedback based antimicrobial stewardship program at a private tertiary care hospital. Indian J Med Microbiol.

[106] Messina AP, van den Bergh D, Goff DA (2015). Antimicrobial stewardship with pharmacist intervention improves timeliness of antimicrobials across thirty-three hospitals in South Africa. Infect Dis Ther.

[107] Sarang B, Tiwary A, Gadgil A, Roy N (2020). Implementing antimicrobial stewardship to reduce surgical site infections: experience and challenges from two tertiary-care hospitals in Mumbai, India. J Glob Antimicrob Resist.

[108] van den Bergh D, Messina AP, Goff DA, van Jaarsveld A, Coetzee R, de Wet Y, Bronkhorst E, Brink A, Mendelson M, Richards GA (2020). A pharmacist-led prospective antibiotic stewardship intervention improves compliance to community-acquired pneumonia guidelines in 39 public and private hospitals across South Africa. Int J Antimicrob Agents.

[109] Li Z, Cheng B, Zhang K, Xie G, Wang Y, Hou J, Chu L, Zhao J, Xu Z, Lu Z (2017). Pharmacist-driven antimicrobial stewardship in intensive care units in East China: a multicenter prospective cohort study. Am J Infect Control.

[110] Zheng N, Li J, Liu Y, Liao K, Chen J, Zhang C, Wen W (2023). Evaluation of implementation and effectiveness of China's antibiotic stewardship in the First Affiliated Hospital of Sun Yat-sen University. Antibiotics (Basel).

[111] Sumon SA, Anwar MMU, Akther FM, Priyanka AS, Tamanna T, Rahman A, Islam MS, Dostogir Harun MG (2023). Perceptions of antibiotic stewardship program and determinants of antibiotic prescribing pattern among physicians in tertiary hospitals in Bangladesh: implications for future policy and practice. J Hosp Infect.

[112] Stanic Benic M, Milanic R, Monnier AA, Gyssens IC, Adriaenssens N, Versporten A, Zanichelli V, Le Maréchal M, Huttner B, Tebano G (2018). Metrics for quantifying antibiotic use in the hospital setting: results from a systematic review and international multidisciplinary consensus procedure. J Antimicrob Chemother.

[113] Karanika S, Paudel S, Grigoras C, Kalbasi A, Mylonakis E (2016). Systematic review and meta-analysis of clinical and economic outcomes from the implementation of hospital-based antimicrobial stewardship programs. Antimicrob Agents Chemother.

[114] Lee CF, Cowling BJ, Feng S, Aso H, Wu P, Fukuda K, Seto WH (2018). Impact of antibiotic stewardship programmes in Asia: a systematic review and meta-analysis. J Antimicrob Chemother.

[115] Baur D, Gladstone BP, Burkert F, Carrara E, Foschi F, Döbele S, Tacconelli E (2017). Effect of antibiotic stewardship on the incidence of infection and colonisation with antibiotic-resistant bacteria and Clostridium difficile infection: a systematic review and meta-analysis. Lancet Infect Dis.

[116] WHO (2019). Antimicrobial stewardship programmes in health-care facilities in low- and middle-income countries: a WHO practical toolkit. JAC Antimicrob Resist.

