# Supplementary material for: Barriers, facilitators, perceptions and impact of interventions in implementing antimicrobial stewardship programs in hospitals of low-middle and middle countries: a scoping review
Source: Antimicrob Resist Infect Control. 2024 Jan 23;13:8. doi: 10.1186/s13756-024-01369-6 (PMC10804809; doi:10.1186/s13756-024-01369-6)
Supplement: Supplementary file 1 — Additional file 1: Appendix 1. Search strategy. [file 13756_2024_1369_MOESM1_ESM.docx]

Additional file 1 (Appendix 1)

Search strategy for Scoping Review

| 1 | (("antimicrobial"[tw] OR "antibiotic"[tw] OR "antimicrobial resistance"[tw] OR "antibiotic resistance"[tw])) |
| --- | --- |
| 2 | (("stewardship"[tw] OR "formulary restriction"[tw] OR "education"[tw] OR "de-escalation therapy"[tw] OR "guidelines"[tw] OR "prospective audit"[tw] OR "drug monitoring"[tw] OR "antibiogram"[tw])) |
| 3 | ((LMIC OR (Low-Middle-Income countries) OR (less developed countries) OR underdeveloped OR Developing Countries OR developing countr* OR ("developing country" OR "developing countries" OR "developing nation" OR "developing nations" OR "developing population" OR "developing populations" OR "developing world" OR "less developed country" OR "tinct " OR "less developed nation" OR "less developed nations" OR "less developed world" OR "lesser developed countries" OR "under developed country" OR "under developed countries" OR "under developed nations" OR "under developed world" OR "underdeveloped country" OR "underdeveloped countries" OR "underdeveloped nation" OR "underdeveloped nations" OR "underdeveloped world" OR "middle income country" OR "middle income countries" OR "middle income nation" OR "middle income nations" OR "middle income population" OR "middle income populations" OR "low income country" OR "low income countries" OR "low income nation" OR "low income nations" OR "low income population" OR "low income populations" OR "lower income country" OR "lower income countries" OR "lower income nations" OR "lower income population" OR "lower income populations" OR "underserved population" OR "underserved populations" OR "under served population" OR "under served populations" OR "deprived countries" OR "deprived population" OR "deprived populations" OR "poor country" OR "poor countries" OR "poor nation" OR "poor nations" OR "poor population" OR "poor populations" OR "poor world" OR "poorer countries" OR "poorer nations" OR "poorer population" OR "poorer populations" OR "developing economy" OR "developing economies" OR "less developed economy" OR "less developed economies" OR "underdeveloped economy" OR "underdeveloped economies" OR "middle income economy" OR "middle income economies" OR "low income economy" OR "low income economies" OR "lower income economies" OR "low GDP" OR "low gross domestic" OR "low gross national" OR "lower GDP" OR "lower gross domestic" OR LMIC OR LMICs OR "third world" OR "LAMI country" OR "LAMI countries" OR "transitional country" OR "transitional countries" OR Africa OR Asia OR Caribbean Region OR West Indies OR South America OR Latin America OR Central America OR Afghanistan OR Albania OR Algeria OR Angola OR Argentina OR Armenia OR Armenian OR Azerbaijan OR Bangladesh OR Benin OR Byelarus OR Byelorussian OR Belarus OR Belorussian OR Belorussia OR Belize OR Bhutan OR Bolivia OR Bosnia OR Herzegovina OR Hercegovina OR Botswana OR Brazil OR Bulgaria OR Burkina Faso OR Burkina Fasso OR Upper Volta OR Burundi OR Urundi OR Cambodia OR Khmer Republic OR Kampuchea OR Cameroon OR Cameroons OR Cameron OR Camerons OR Cape Verde OR Central African Republic OR Chad OR China OR Colombia OR Comoros OR Comoro Islands OR Comores OR Mayotte OR Congo OR Zaire OR Costa Rica OR Cote d'Ivoire OR Ivory Coast OR Cuba OR Djibouti OR French Somaliland OR Dominica OR Dominican Republic OR East Timor OR East Timur OR Timor Leste OR Ecuador OR Egypt OR United Arab Republic OR El Salvador OR Eritrea OR Ethiopia OR Fiji OR Gabon OR Gabonese Republic OR Gambia OR Gaza OR Georgia Republic OR Georgian Republic OR Ghana OR Gold Coast OR Grenada OR Guatemala OR Guinea OR Guiana OR Guyana OR Haiti OR Honduras OR India OR Maldives OR Indonesia OR Iran OR Iraq OR Isle of Man OR Jamaica OR Jordan OR Kazakhstan OR Kazakh OR Kenya OR Kiribati OR Korea OR Kosovo OR Kyrgyzstan OR Kirghizia OR Kyrgyz Republic OR Kirghiz OR Kirgizstan OR "Lao PDR" OR Laos OR Lebanon OR Lesotho OR Basutoland OR Liberia OR Libya OR Macedonia OR Madagascar OR Malagasy Republic OR Malaysia OR Malaya OR Malay OR Sabah OR Sarawak OR Malawi OR Nyasaland OR Mali OR Marshall Islands OR Mauritania OR Mauritius OR Agalega Islands OR Mexico OR Micronesia OR Middle East OR Moldova OR Moldovia OR Moldovian OR Mongolia OR Montenegro OR Morocco OR Ifni OR Mozambique OR Myanmar OR Myanma OR Burma OR Namibia OR Nepal OR Nicaragua OR Niger OR Nigeria OR Muscat OR Pakistan OR Palau OR Palestine OR Panama OR Paraguay OR Peru OR Philippines OR Philipines OR Phillipines OR Phillippines OR Romania OR Rumania OR Roumania OR Russia OR Russian OR Rwanda OR Ruanda OR Saint Lucia OR St Lucia OR Saint Vincent OR St Vincent OR Grenadines OR Samoa OR Samoan Islands OR Navigator Island OR Navigator Islands OR Sao Tome OR Senegal OR Serbia OR Montenegro OR Seychelles OR Sierra Leone OR Sri Lanka OR Ceylon OR Solomon Islands OR Somalia OR Sudan OR Suriname OR Surinam OR Swaziland OR Syria OR Tajikistan OR Tadzhikistan OR Tadjikistan OR Tadzhik OR Tanzania OR Thailand OR Togo OR Togolese Republic OR Tonga OR Tunisia OR Turkey OR Turkmenistan OR Turkmen OR Uganda OR Ukraine OR USSR OR Soviet Union OR Union of Soviet Socialist Republics OR Uzbekistan OR Uzbek OR Vanuatu OR New Hebrides OR Venezuela OR Vietnam OR Viet Nam OR West Bank OR Yemen OR Yugoslavia OR Zambia OR Zimbabwe OR Rhodesia)) |
| 4 | 1 AND 2 AND 3 |
| 5 | Limit to “Human”, “2015-2023”, “English” |
